# On the impact of oil compounds on emulsion behavior under different thermodynamic conditions

**DOI:** 10.1038/s41598-023-42886-5

**Published:** 2023-09-21

**Authors:** Milad Mohammadpour, M. Reza Malayeri, Yousef Kazemzadeh, Masoud Riazi

**Affiliations:** 1 Enhanced Oil Recovery Research Center, School of Chemical and Petroleum Engineering, Shiraz University, Shiraz, Iran; 2 Department of Chemical Engineering, School of Chemical and Petroleum Engineering, Shiraz University, Shiraz, Iran; 3 Department of Petroleum Engineering, Faculty of Oil, Gas, and Petrochemical Engineering, Persian Gulf University, Bushehr, Iran; 4 School of Mining and Geosciences, Nazarbayev University, Kabanbay Batyr 53, 010000 Astana, Kazakhstan

**Keywords:** Engineering, Chemical engineering

## Abstract

Asphaltene instability in oil causes severe problems such as deposition and more stable emulsions. Formation and stability of W/O emulsions based on location in which they are formed can either be helpful or detrimental for enhanced oil recovery. Changes in oil composition (saturate, aromatic, resin, and asphaltene) can also render the stability of asphaltene. In this study, the formation and staility of emulsions are investigated using changes in the colloidal instability index (CII) at ambient and reservoir conditions. Experiments were conducted for crude oil samples from various reservoirs which showed that when CII is greater than 1.059, due to the excessive instability of asphaltene and its movement toward the water–oil interface, the formed emulsion would be more stable. When CII was below 1.059 though, the asphaltene became stable hence did not tend to be placed at the water–oil interface, thus less stable emulsion was expected. Higher pressures led to an increase in the stability of the emulsion. These changes in the process of emulsion stability are related to two mechanisms of asphaltene absorption and greater shear stresses.

## Introduction

Many studies have addressed the subject of oil and water emulsions in recent years. Asphaltene has the ability to stabilize the emulsion because of its moderate capacity to reduce the interfacial tension between water and oil^1^. McLean and Kilpatrick (1997) showed that asphaltene plays a key role in the stability of W/O emulsion. They also observed that asphaltene shows ability to reduce the water–oil interfacial tension and stabilize the emulsion.

The ingredients and their concentration in emulsion directly affects its stability. Another important factor that influences the emulsion stability is the particle size. A minimal amount of material and particles is required to form an emulsion^2,3^. Goldszal et al. (2002) studied the effect of naphthenates on the emulsion stability and showed that the emulsion stability of water in acidic oil is higher than less acidic oil^4^.

Asphaltene is the heaviest and most polar constituent in crude oil. It would be insoluble in low molecular weight alkanes while it would be soluble in aromatics such as toluene and benzene^5^. One of the most important causes of asphaltene precipitation in oil is pressure changes and the injection of solvents during enhanced oil recovery as well as operating conditions^6^.

Monitoring the stability of asphaltene in crude oil is still a matter of much debate hence has been studied extensively^7^. The simultaneous presence of asphaltene and fractions of resins, saturates and aromatics in oil has a profound impact on the formation and stabilization of emulsion^8^. Asphaltene has the ability to make a stable emulsion due to the reduction of interfacial tension between oil and water^1,9,10^. Asphaltene influences the emulsion stability before aggregation. Near the starting point of aggregation, it plays an important role, after that, asphaltene becomes unstable, which causes less influence on emulsion stability^11,12^.

As the concentration of asphaltene increases, the hydrogen bond formed between the asphaltene and the water molecules decrease the interfacial tension between water and oil^13^. Tchoukov et al.^14^ investigated a type of emulsion with drainage kinetic analysis and were able to measure the thickness of the asphaltene layer between water and oil. They found that the equilibrium layer was 10–50 nm thick^15^. The composition of crude oil consists of aliphatic, aromatic hydrocarbons, oxygen, nitrogen, and sulfur, in addition to resin and asphaltene compounds. Many studies showed that the mechanism of forming water in oil emulsion in the presence of asphaltene is through the formation of a viscous film with a high-strength three-dimensional network^16,17^.

Contrary to oil samples containing a high concentration of asphaltene, in some samples, no asphaltene precipitation has been observed. This was due to thermodynamic conditions, such as temperature, pressure, as well as oil compounds^18^. More asphaltene precipitation is not related to higher asphaltene content, but rather to saturate and resin contents. Increasing non-polar components, such as saturates, increases oil instability, i.e. more saturates means higher asphaltene precipitation^19^. Fan et al. (2010) found that at a constant concentration of asphaltene, increasing the solubility of aromatics reduced the elastic strength of the film. As asphaltene content increases, the emulsion becomes more stable. According to microscopic imaging, the size of water droplets decreased when the asphaltene content increased, and the emulsion became unstable when the asphaltene content reached zero^20^.

The addition of toluene to oil reduces the size of the asphaltene aggregates. Previous studies have shown that the most stable emulsions are formed with more oil and asphaltene in the system^21^. Furthermore, increasing the water content in the emulsion led to changes in the droplet size and droplet size distribution, which resulted in more asphaltene precipitation. In organic solutions containing less toluene, a more stable emulsion is expected^22^. The presence of asphaltene in the emulsion structure can make it stable. With the addition of BR bonding resins, stability was reduced as these resins prevent emulsions from being stabilized by asphaltene^23^.

In addition to increased pressure to an optimal level, increasing the shear stress also leads to smaller water droplets in the oil phase, which results in more stable water in oil emulsion as well as a reduction in the interfacial tension between the two phases. After the optimum pressure, further increase in pressure led to less stable emulsion due to decrease in the amount of asphaltene precipitation at the interface of water and oil. Under such circumstances, the size of water droplets increased, and the interfacial tension between the water and oil increased^24^(Shams et al., 2022).

The aim of this study was to investigate the behavior of W/O emulsions in the presence of asphaltene. For this purpose, the colloidal instability index (CII), which indicates the degree of asphaltene instability, was changed by adding a representative of saturates and aromatics, which can affect the behavior of emulsions. These tests were then compared at ambient and high-pressure conditions. The main difference between this investigation and other studies is that all experiments were performed at high-pressure conditions using the HTHP device. Different test conditions also affected the behavior of asphaltene in oil, which in turn influenced thr formation and stability of emulsions.

## Materials and methods

### Oil and water

In this study, oil was obtained from one of the oil fields in south of Iran. The average temperature and pressure in this reservoir were 245° F and 4602 psi, respectively. The measured viscosity at 80.6 ℉ was 391 cp. The API gravity and oil density were also 19 and 0.94 g/cm^3^, respectively. Initially, the oil was centrifuged at 7000 RPM for 10 min to remove any water in the oil phase to ensure that there was no water in the oil phase. Distilled water was used as the aqueous phase.

### SARA and colloidal instability index(CII)

**Figure 1:**
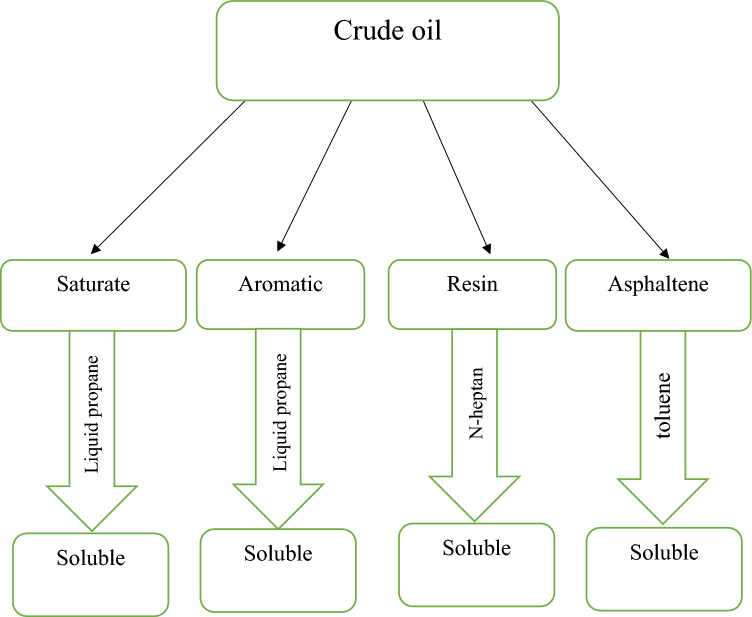
Flowchart of SARA analysis.

SARA analysis of the crude oil was performed using the modified method IP143 of which the results are presented in Table 1. With the help of this test the amounts of asphaltene, resin, saturate, and aromatic compounds were obtained. The colloidal instability index (CII) can be defined using data obtained from SARA analysis. Figure 1 shows a flowchart of the SARA analysis. The solubilities of these components are specified in this flowchart. For example, if an oil sample is added to light alkanes, the aromatics and saturates would be dissolved, but asphaltene and resin precipitate. Thus, the weight percentage of each component can be obtained^25^.

**Table 1:** Properties of the investigated crude oil.

Component	Wt%
Saturates	43.4
Aromatics	35.6
Resins	12.9
Asphaltenes	8
TBN, mgKOH/g	1.93
TAN, mgKOH/g	2.03

The colloidal instability index considers crude oil as a colloidal system containing asphaltene, saturates, resins, and aromatics. The colloidal instability index is the ratio of the sum of asphaltene and saturates to the sum of aromatics and resins, which can be used as a measure to characterize the stability or instability of asphaltene in crude oil.

1 $$\mathrm{CII}=\frac{\mathrm{ Asphaltenes}+\mathrm{Asaturates}}{\mathrm{Aromatics}+\mathrm{Resins}}$$

An oil with a CII below 0.7 is stable, and over 0.9 is considered highly unstable. If the CII is between 0.7 and 0.9, oil has moderate stability^26^.

### Saturate and aromatic representatives

To better understand the behavior of water-in-oil emulsions, changes were made to the crude oil compositions, which were applied to the colloidal instability index (CII). In this study, n-heptane was used as saturate agent and toluene as aromatic.

### Experimental setup

**Figure 2:**
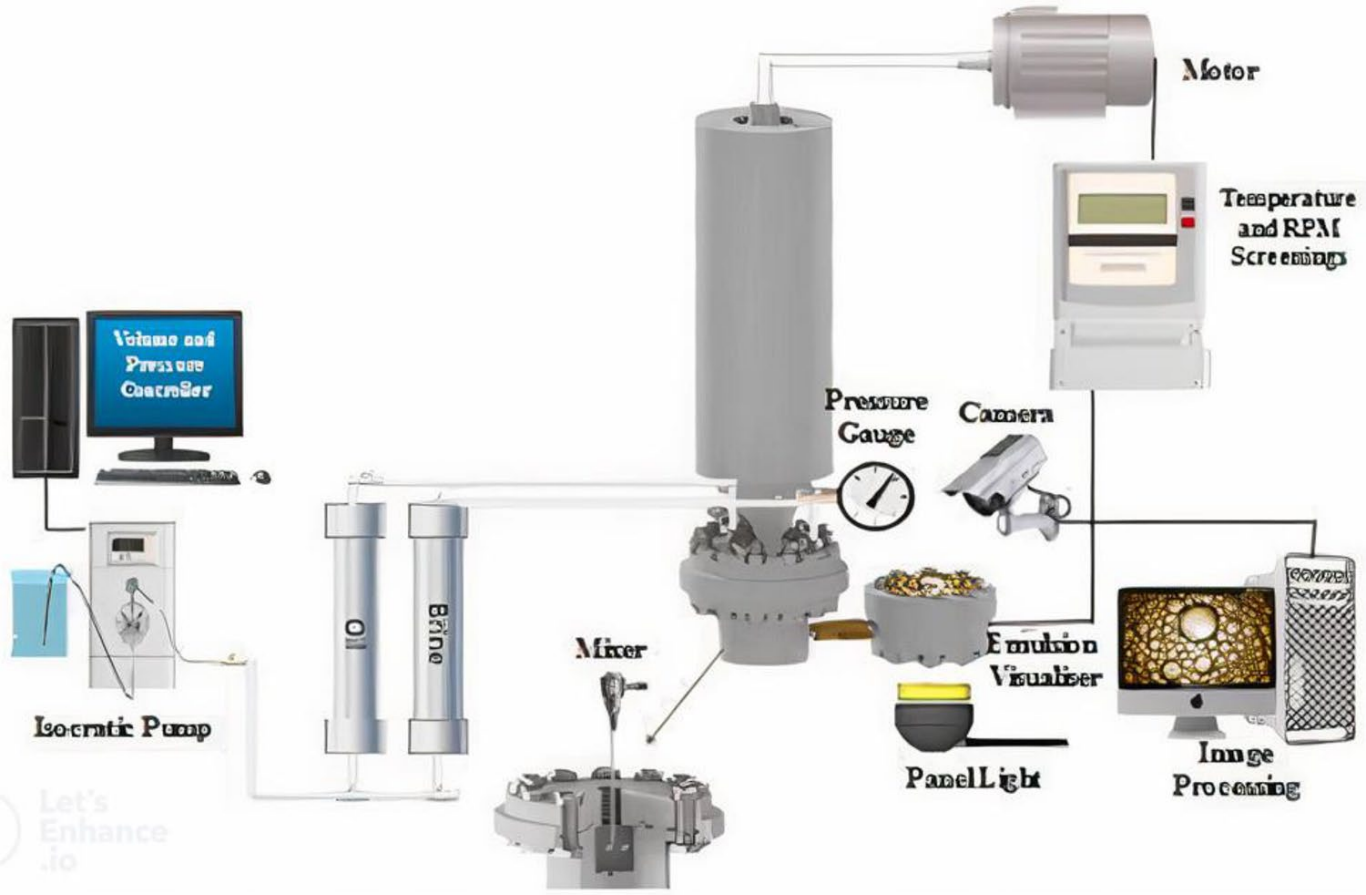
General schematic of measuring device formation and stability of emulsion at high temperature and pressure^24^.

An apparatus, as depicted in Fig. 2^24^, was used to assess the stability of emulsions under reservoir conditions. Initially, to form an emulsion, oil and water were pumped from two separate cylinders using an Agilent pump at a specific flow rate. Subsequently, a strong mechanical stirrer was used with a rotational speed of 4000 RPM to mix the two phases. In other words, the engine of the stirrer transfers the required torque to the blades embedded in the fluid chamber. Subsequently, the sample was ready for the visual chamber at specified pressure and temperature. The sample was then placed on a screen before taking images by a microscope by opening the middle valve. The size distribution of water droplets in the oil was examined by image analysis using the ImageJ software. The mechanism of emulsion formation in this device involves the use of shear force. Shear energy causes the droplets to become smaller, thereby increasing the surface area of the emulsion, which leads to an increase in the number of droplets. Other devices, such as homogenizers and ultrasonic and magnetic stirrers, can also provide the required energy to reduce the size of water droplets. The shear energy from the stirrer and homogenizer to reduce the size of water droplets to micro size is from 500 to 12,000 rpm and when ultrasonic is used, the size of water droplets is usually reduced to nano sizes. The emulsion stability depends on various factors, such as the strength of the interface between oil and water, water droplet sizes, average area of the water droplets, that change over time. In order to calculate the average surface area of water droplets and their size distribution, the ImageJ software was used Thereafter, centrifuge was used to separate the two phases of water and oil and also to determine the semulsion tability.

Emulsions can be formed with different stabilities at both ambient and reservoir conditions. One of the ways to check the stability of an emulsion is to use a centrifuge. This stability study was based on the energy input from the centrifuge to separate the two phases of water and oil. In the experiments, model 200 of Hettich centrifuge with a maximum speed of 13,000 RPM was used.

#### Emulsion preparation at ambient condition

To measure and evaluate the stability of water in oil emulsion, 70 vol% oil and 30 vol% water were used. These percentages have been used for two reasons. The first reason was to investigate the effect of water spread for five different volume percentages, which the most stable was at 30 vol% of water. The second reason is that this percentage was close to real conditions of the reservoir. All fluid measurements were performed using a graduated cylinder. For adding the aqueous phase to the oil, it was placed in a magnetic stirrer at 500 RPM before slowly adding water in dropwise manner for 15 min. To investigate the effect of oil composition on emulsion formation and stability, six oil samples with different CII values were also prepared by adding a certain amount of n-heptane and toluene. The weight percentage of each component of the prepared samples in 30 g of oil are given in Table 2.

**Table 2:** Weight percentage of crude oil components.

N-heptane (wt%)	Asphaltene (wt%)	Resins (wt%)	Toluene (wt%)	Oil sample	CII
47.00	7.40	12.00	33.33	M1	1.5
49.16	7.18	11.58	31.97	M2	1.3
53.35	6.59	10.63	29.33	M3	1.2
43.4	8.00	12.90	35.6	M4	1.059
39.96	7.36	11.58	40.69	M5	0.9
34.74	6.40	10.32	48.44	M6	0.7
28.12	5.18	8.36	58.26	M7	0.5

#### Emulsion preparation at reservoir condition

To prepare a W/O emulsion and investigate the effect of oil composition on the formation and stability of emulsions, the high temperature and pressure device described in the previous section was used. The volume of oil was 70 vol% and water 30 vol%, and toluene and n-heptane (Table 2) per 200 gr of base oil have been used to change the colloidal instability index. At the beginning, the desired temperature for the experiments was 80 °C; however, due to a lack of emulsion formation at this temperature, it was decided to reduce the temperature to 27 °C. The pressure in this study was 3500 psi. After adding water slowly into the oil phase, the mixer was then turned on, and the water and oil were mixed for 10 min at a specific pressure.

### W/O droplet size distribution

The droplet size distribution was used to analyze the stability of formed emulsions. After preparing the emulsion at ambient conditions and HTHP apparatus, the emulsion was sampled using a syringe and analyzed by an AMS-PZ200TB microscope. In order to ensure the accuracy of the data, several samples were taken, followed vy the calculation of droplet size distribution using the ImageJ software. In order to better analyze and compare the images taken from the microscope, the magnification and scale of all images were similar. To better identify the droplets, a threshold was set for the images to detect the droplet size distribution more accurately. Once this was accomplished then the droplets turned black while the other components of the image became white. The image was then ready for analysis of the droplet size distribution.

### Phase separation test

After analyzing the droplet size distribution of emulsions, another method used to evaluate emulsions' stability based on phase separation at different RPMs by centrifugal force with the centrifuge. In this analysis, samples at different conditions, including pressure and CII, were poured into the calibrated 10 ml cylinders and placed in a centrifuge. It started spinning at low speeds, and when 20 vol% of the water in the emulsion separated into the container, then the rotation speed was considered as the final RPM. In order to ensure the reliability and repeatability of the tests, all tests were repeated twice of which their errors have been specified in the following respective figures.

## Results and discussion

### Emulsion formation and stability at ambient condition

**Figure 3:**
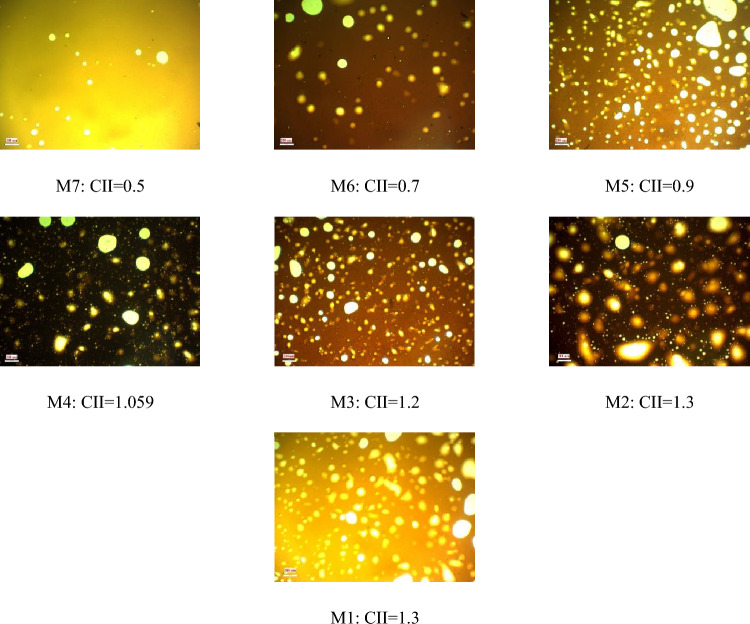
Microscopic image of the emulsion under ambient (25℃, 14.6 psi) conditions (Scale for all photos 200 µm).

**Figure 4:**
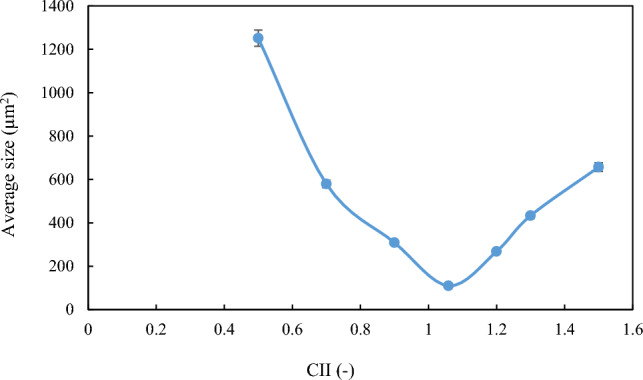
Average surface area of droplets (μm^2^)in oil under ambient conditions.

As explained in the previous sections, the formation and stability of W/O emulsions depend on various factors, including, but not limited to, oil-phase and aqueous-phase compounds, as well as thermodynamic conditions such as pressure and temperature. To proceed, firstly, the relationship between aromatics/saturates with the colloidal instability index was examined. Compared with other parts of the oil, aromatics can also be polarizable. Asphaltene stability depends on the relative proportion between the SARA fractions. The presence of aromatic rings in oil next to asphaltene, which has a polar surface, can help the stability of asphaltene in oil. In contrast, saturates in oil are known as a non-polar components, and the higher percentage of saturates corresponds to more oil instability. According to previous studies, oil samples with high saturates and less asphaltene are more vulnerable to asphaltene precipitation than oils with lower saturates and higher asphaltene content^19^. Nanoscale asphaltene aggregates can activate the oil surface and stabilize the W/O emulsion while still being soluble in the solvent. After preparing the emulsion using the method described in the methodology section, image recording was started using a microscope, before calculating the droplet size distribution. Microscopic images of samples made with different colloidal instability indices are shown in Fig. 3. A diagram of the average size of the water droplets in the oil is shown in Fig. 4. As shown in this figure, the most stable emulsion, formed under ambient conditions, is the M4 sample, with a colloidal instability index of 1.059, which is similar to that of crude oil. On the other hand, the addition of toluene to crude oil reduces the colloidal instability index (CII). Having considered that aromatics are suitable solvents for asphaltene in oil, then they serve as emulsifier (natural surfactant) hence would reduce the stability of the formed emulsion. In other words, by adding toluene to crude oil, the asphaltene in the crude oil becomes more stable and suspended, and a small amount of asphaltene would tend to be trapped at the interface of oil and water to form an emulsion film. This would then reduce the amount of emulsifying agent. In the second stage of the tests, the addition of n-heptane as representative of saturates to crude oil increased the oil colloidal instability index, which caused the instability of asphaltene in crude oil. This, in turn, caused a better placement of asphaltene at the interface between water and oil causing a more stable emulsion compared with when using toluene^7,24,27,28^.

Previous studies have shown that asphaltenes may bind tightly to each other and the interface layer through intermolecular interactions, such as π bonding between aromatic groups, charge transfer interactions, van der Waals interactions, hydrogen bonds, and multipolar forces, resulting in the elasticity of the asphaltene layers and emulsion stability^3,29,30^.

It is worth mentioning that by adding too much n-heptane to crude oil, asphaltenes in crude oil become unstable and form asphaltene clusters that change with time and thermodynamic conditions. They would gradually precipitate and when this occurs, then asphaltene will not be able to be present at the interface of water and oil. For this reason, there will be no other emulsifying substances that affect the stability of the emulsion. However, the stability of emulsions with a colloidal instability index greater than 1.059 is higher than that of emulsions with values less than 1.059 owing to the presence of asphaltene stabilizers, i.e. aromatics.

**Figure 5:**
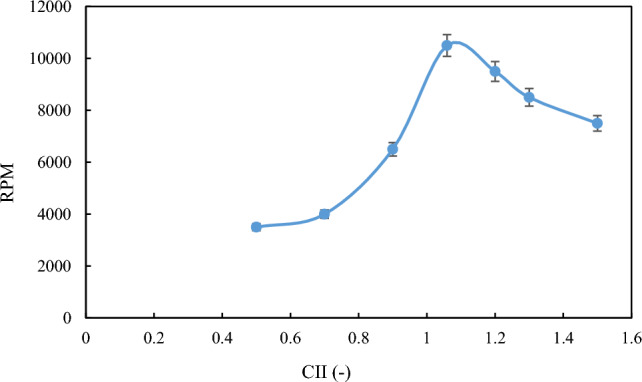
Variation of RPM with CII for separation of water phase from oil under ambient conditions.

**Figure 6:**
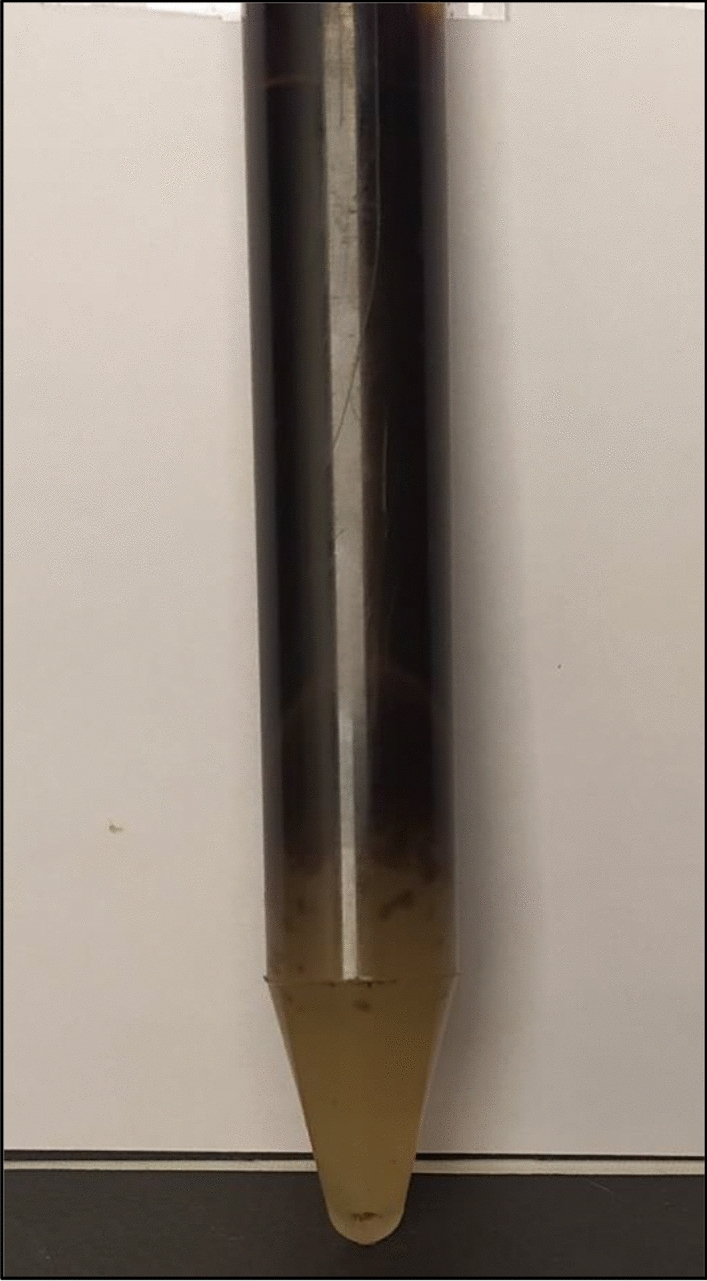
Separation of two phases in the centrifuge.

Another method to evaluate the stability of the formed emulsion is to investigate the separation of the two phases using a centrifuge. Table 3 implies that the separation of the aqueous phase from the oil phase in each of the formed emulsions is very consistent with the droplet size distribution results. Owing to increased emulsion stability, two-phase separation occurred at higher RPM. Therefore, a higher rotational speed is required to achieve a two-phase separation. Figure 5 shows the RPM diagram of the separation of aqueous phase from oil, and Fig. 6 shows an example of this phase separation by the centrifuge.

**Table 3:** RPM for separation of water phase from oil under ambient conditions.

CII	Oil sample	RPM
0.5	M1	3500
0.7	M2	4000
0.9	M3	6500
1.059	M4	10,500
1.2	M5	9500
1.3	M6	8500
1.5	M7	7500

### Emulsion formation and stability at high pressure

In this part, the formation and stability of emulsions at high pressure conditions would be presented. As mentioned in Section "Emulsion preparation at reservoir condition", the pressure used to perform the tests was 3500 psi. The process of performing the tests was the same as those at the ambient conditions; that is, by adding toluene and n-heptane, the impact of changes in composition of crude oil has been examined. Given the HTHP device and the volume of the main chamber in which the fluid is placed, then more fluid is required than that under ambient conditions. The operation of this device is described in Section "Results and discussion". All samples were subjected to a pressure of 3500 psi, and the mechanical force required to form the emulsion was applied for 10 min by turning on the mechanical blade installed in the device. Subsequently, microscopic images were taken from the prepared samples, and the droplet size distribution and average droplet surface area were then calculated.

**Figure 7:**
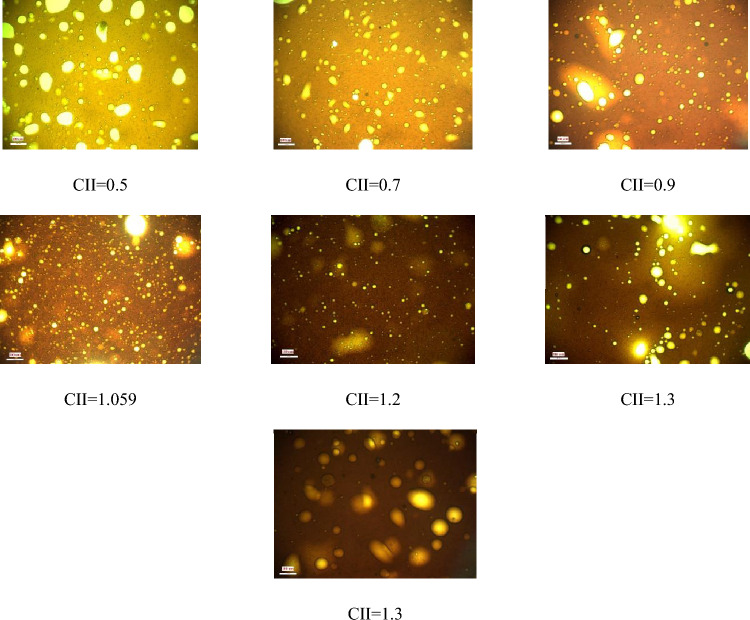
Microscopic images of emulsions at high pressure conditions (3500 psi) (Scale for all photos 100 µm).

In general, two factors must exist so that an emulsion can be formed and remain stable namely (i) mechanical energy and (ii) chemical energy. Mechanical energy is applied to a fluid by the shear energy of blades, which operates at high and low pressures. Chemical energy refers to the presence of a surfactant, here asphaltene. As a natural surfactant in crude oil, asphaltene is an important factor for the formation and stability of emulsions. Asphaltene has a higher polarity than the other components of crude oil because of the presence of heteroatoms such as oxygen, nitrogen, sulfur, and heavy metals in its structure. Therefore, asphaltene molecules tend to be placed at the interface of water and oil phases to form stable emulsions^31,32^. As shown in Fig. 7, as the pressure increased to 3500 psi, the droplet size decreased and becomes smaller. It can be inferred that the stability of emulsions formed at the elevated pressure is then higher than those at ambient pressure. Moreover, the average size of water droplets in the emulsion would be decreased, which is attributed to the absorption mechanism (asphaltene presence at the W/O interface) and shear force. As pressure increased, the amount of asphaltene precipitated between the water and oil decreases, causing it to become chemically unstable. Another mechanism that leads to the formation of emulsions and the shrinkage of water droplets is the increase of shear energy.

**Figure 8:**
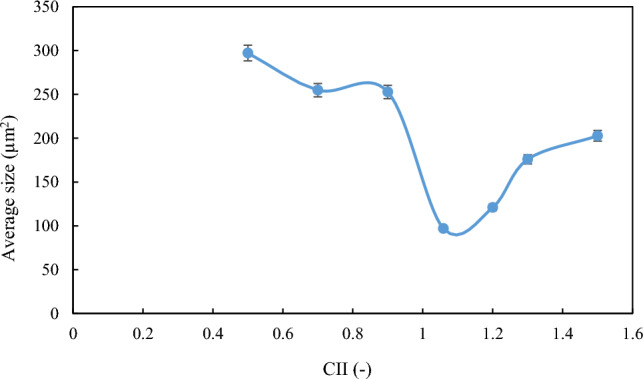
Average surface area of droplets (μm^2^) in oil at high pressure conditions.

As pressure increased, the amount of shear energy applied by the mechanical blades into the chamber increased, and the size of the oil droplets decreased, resulting in a more stable emulsion^7,24,33^. Despite the two mechanisms of mechanical and chemical energy, at a pressure of 3500 psi, mechanical energy prevails over chemical energy and causes droplet size to decrease. As the emulsion became more stable, the water droplets became finer. The average level of water droplets in the oil at a pressure of 3500 psi when toluene and heptane were added is shown in Fig. 8.

**Figure 9:**
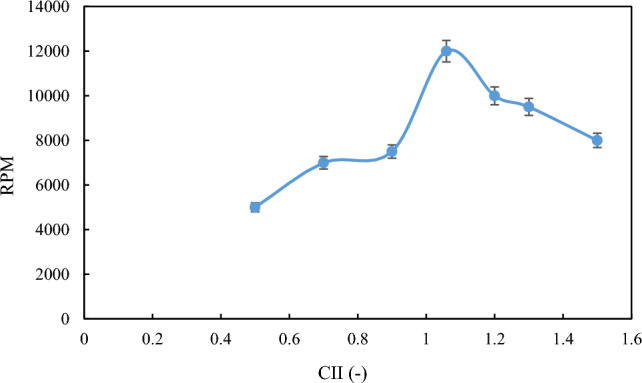
RPM diagram for separation of water phase from oil under high-pressure conditions.

At ambient conditions, the method of two-phase separation was used to evaluate the stability of emulsions formed at high pressures. By placing the samples prepared at different conditions inside the centrifuge and using different RPM, the separation of the two phases for each sample from each other at a given RPM is provided in Table 4, as well as Fig. 9.

**Table 4:** The required RPM for separation of water from oil under high pressure conditions.

CII	Oil sample	RPM
0.5	M1	5000
0.7	M2	7000
0.9	M3	7500
1.059	M4	12,000
1.2	M5	10,000
1.3	M6	9500
1.5	M7	8000

### Comparison of thermodynamic conditions for stability of emulsions

**Figure 10:**
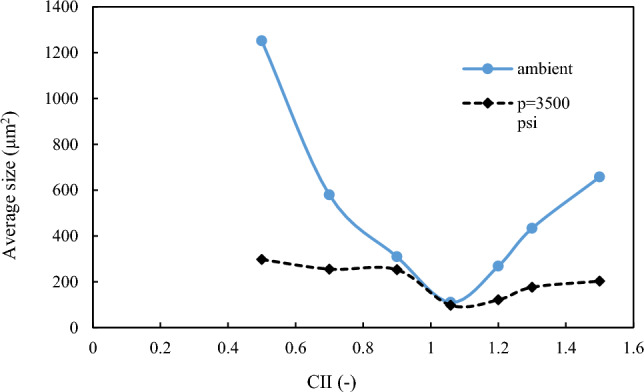
Comparison between the stability of emulsions formed under ambient and high pressure conditions.

According to Fig. 10, the average size of water droplets is lower at high pressures compared to that at ambient conditions. This implies that the formed emulsions are more stable at higher pressure. The degree of stability and emulsion formation for the M4 sample (CII = 1.059) at both ambient and high pressure conditions are close to each other. As mentioned in the previous section, the cause of emulsion formation is the instability of asphaltene, and according to previous studies, when the colloidal instability index is between 0.9 and 0.7, then it is relatively stable, and when less than 0.7^34^, it is stable and the emulsion would be unstable when CII is greater than 0.9. At 1.059, asphaltene was unstable when no substance was added to the oil. The asphaltene instabilities under ambient and high pressure conditions were close to each other, resulting in relatively equal stabilities of the two emulsions. The difference in droplet sizes under ambient and high pressure conditions can be related to the amount of asphaltene in the colloidal instability index. With the addition of toluene, the asphaltene was stabilized and dispersed in the oil phase. This, in turn, reduced the amount of asphaltene at the oil–water interface thus a less stable emulsion should be expected. The reason for the difference in slope between the two lines of the diagram around point 1.059 at ambient and high pressure conditions is the dominance of respective mechanism affecting the stability of the emulsion. As pressure increased, the exerted mechanical energy would be increased from that at ambient conditions, which made the water droplets finer and emulsion more stable. The difference in slope between the two side edges is the amount of mechanical energy applied to the emulsion.

## Conclusions

The primary purpose of this study was to investigate the effects of oil compounds under different thermodynamic conditions on the formation and stability of W/O emulsions. Various factors that would affect the formation of emulsions including the thermodynamic conditions, and oil composition were investigated. In this study, asphaltene acted as a natural emulsifier and surfactant thus the stability of asphaltene would govern the behaviour of emulsion. For this purpose, using heptane and toluene as representatives of saturates and aromatics, six oil samples were prepared with six different colloidal instability indices. The results showed that the most stable emulsion was the M4 sample and the most unstable emulsion was the M1 sample under both ambient and high pressure conditions. In addition, the results showed that by increasing the pressure near the bubble point, the asphaltene in the crude oil became unstable, making the W/O emulsion more stable. At the elevated pressure, more asphaltene was available at the interface of water and oil due to lower solubility of asphaltene,. This makes the W/O emulsion more stable at high pressures than that at the ambient conditions.

## Data Availability

All data generated or analysed during this study are included in this published article.

## References

[1] McLean JD, Kilpatrick PK (1997). Effects of asphaltene aggregation in model heptane-toluene mixtures on stability of water-in-oil emulsions. J. Colloid Interface Sci..

[2] Perles CE, Volpe PLO, Bombard AJF (2012). Study of the cation and salinity effect on electrocoalescence of water/crude oil emulsions. Energy Fuels.

[3] Ma J, Yang Y, Li X, Sui H, He L (2021). Mechanisms on the stability and instability of water-in-oil emulsion stabilized by interfacially active asphaltenes: Role of hydrogen bonding reconstructing. Fuel.

[4] Goldszal, A., Hurtevent, C., & Rousseau, G. Scale and naphthenate inhibition in deep-offshore fields. In *SPE International Oilfield Scale Conference and Exhibition?*, pp. 107–117 (2002). 10.2118/74661-ms.

[5] Rodr, S., Torres-mancera, P., & Ancheyta, J. Evaluation of asphaltene stability of a wide range of mexican crude oils (2021). 10.1021/acs.energyfuels.0c03301.

[6] Alizadeh A, Nakhli H, Kharrat R, Ghazanfari MH (2011). An experimental investigation of asphaltene precipitation during natural production of heavy and light oil reservoirs: The role of pressure and temperature. Pet. Sci. Technol..

[7] Ashoori S, Sharifi M, Masoumi M, Mohammad Salehi M (2017). The relationship between SARA fractions and crude oil stability. Egypt. J. Pet..

[8] Andersen SI, Speight JG (2001). Petroleum resins: Separation, character, and role in petroleum. Pet. Sci. Technol..

[9] Ashoorian S, Javadi A, Hosseinpour N, Husein M (2021). Evolution of adsorbed layers of asphaltenes at oil-water interfaces: A novel experimental protocol. J. Colloid Interface Sci..

[10] Chu Y (2023). Experimental study on self—Emulsification of shale crude oil by natural emulsifiers. J. Dispers. Sci. Technol..

[11] Kamkar M, Natale G (2021). A review on novel applications of asphaltenes: A valuable waste. Fuel.

[12] Jagadisan, A. Asphaltene interactions observed at interfaces: A mini review (2023). 10.26434/CHEMRXIV-2023-41SJN.

[13] Moud AA (2022). Asphaltene induced changes in rheological properties: A review. Fuel.

[14] Tchoukov P (2014). Role of asphaltenes in stabilizing thin liquid emulsion films. Langmuir.

[15] Doryani H, Malayeri MR, Riazi M (2016). Visualization of asphaltene precipitation and deposition in a uniformly patterned glass micromodel. Fuel.

[16] Spiecker PM, Kilpatrick PK (2004). Interfacial rheology of petroleum asphaltenes at the oil–water interface. Langmuir.

[17] Sun X, Zeng H, Tang T (2023). Molecular simulations on the coalescence of water-in-oil emulsion droplets with non-ionic surfactant and model asphaltene. Langmuir.

[18] Leontaritis, K. J. Asphaltene deposition: A comprehensive description of problem manifestations and modeling approaches. In *Soc. Pet. Eng. - SPE Prod. Oper. Symp. POS 1989*, 599–613, (1989). 10.2523/18892-ms.

[19] Siddiqui, M. A., Tariq, S. M., Haneef, J., Ali, S. I., & Manzoor, A. A. Asphaltene stability analysis for crude oils and their relationship with asphaltene precipitation models for a gas condensate field. In *SPE Middle East Oil and Gas Show and Conference *MEOS, Proc., vol. 2019-March, (2019). 10.2118/194706-ms.

[20] Uetani T, Kai J, Hitomi T, Seino H, Shimbori K, Yonebayashi H (2020). Experimental investigation of crude-oil emulsion stability: Effect of oil and brine compositions, asphaltene, wax, toluene insolubles, temperature, shear stress, and water cut. SPE Prod. Oper..

[21] Mohammadreza Shams S, Kazemzadeh Y, Riazi M, Cortés FB (2022). Effect of pressure on the optimal salinity point of the aqueous phase in emulsion formation. J. Mol. Liq..

[22] Velayati A, Nouri A (2021). Role of asphaltene in stability of water-in-oil model emulsions: The effects of oil composition and size of the aggregates and droplets. Energy Fuels.

[23] Zargar M, Fridjonsson EO, Graham BF, May EF, Johns ML (2019). Oil-based binding resins: Peculiar water-in-oil emulsion breakers. Energy Fuels.

[24] Ismail I, Kazemzadeh Y, Sharifi M, Riazi M, Malayeri MR, Cortés F (2020). Formation and stability of W/O emulsions in presence of asphaltene at reservoir thermodynamic conditions. J. Mol. Liq..

[25] Soorghali F, Zolghadr A, Ayatollahi S (2014). Effect of resins on asphaltene deposition and the changes of surface properties at different pressures: A microstructure study. Energy Fuels.

[26] Yen, A., Yin, Y. R., & Asomaning, S. Evaluating asphaltene inhibitors: laboratory tests and field studies. In *SPE International Conference on Oilfield Chemistry?*. no. Cii, 613–619, (2001). 10.2118/65376-ms.

[27] Kazemzadeh Y, Ismail I, Rezvani H, Sharifi M, Riazi M (2019). Experimental investigation of stability of water in oil emulsions at reservoir conditions: Effect of ion type, ion concentration, and system pressure. Fuel.

[28] Abdulredha MM, Hussain SA, Abdullah LC, Hong TL (2022). Water-in-oil emulsion stability and demulsification via surface-active compounds: A review. J. Pet. Sci. Eng..

[29] Dehaghani AHS, Taleghani MS, Badizad MH, Daneshfar R (2019). Simulation study of the Gachsaran asphaltene behavior within the interface of oil/water emulsion: a case study. Colloids Interface Sci. Commun..

[30] Kilpatrick PK (2012). Water-in-crude oil emulsion stabilization: Review and unanswered questions. Energy Fuels.

[31] Spiecker PM, Gawrys KL, Trail CB, Kilpatrick PK (2003). Effects of petroleum resins on asphaltene aggregation and water-in-oil emulsion formation. Colloids Surf. A Physicochem. Eng. Aspects.

[32] Shojaati F, Mousavi SH, Riazi M, Torabi F, Osat M (2017). Investigating the effect of salinity on the behavior of asphaltene precipitation in the presence of emulsified water. Ind. Eng. Chem. Res..

[33] Kazemzadeh Y, Shojaei S, Riazi M, Sharifi M (2019). Review on application of nanoparticles for EOR purposes: A critical review of the opportunities and challenges. Chin. J. Chem. Eng..

[34] Salehpour M, Sakhaei Z, Salehinezhad R, Mahani H, Riazi M (2020). Contribution of water-in-oil emulsion formation and pressure fluctuations to low salinity waterflooding of asphaltic oils: A pore-scale perspective. J. Pet. Sci. Eng..

